# Hypothalamic Neuromodulation of Hypothermia in Domestic Animals

**DOI:** 10.3390/ani14030513

**Published:** 2024-02-04

**Authors:** Daniel Mota-Rojas, Marcelo Daniel Ghezzi, Ismael Hernández-Ávalos, Adriana Domínguez-Oliva, Alejandro Casas-Alvarado, Pamela Anahí Lendez, María Carolina Ceriani, Dehua Wang

**Affiliations:** 1 Neurophysiology, Behavior and Animal Welfare Assessment, DPAA, Universidad Autónoma Metropolitana (UAM), Mexico City 04960, Mexico; 2 Animal Welfare Area, Faculty of Veterinary Sciences (FCV), Universidad Nacional del Centro de la Provincia de Buenos Aires (UNCPBA), GIB, Tandil 7000, Buenos Aires, Argentina; 3 Clinical Pharmacology and Veterinary Anesthesia, Biological Sciences Department, FESC, Universidad Nacional Autónoma de México, Cuautitlán 54714, Mexico; 4 Anatomy Area, Faculty of Veterinary Sciences, Universidad Nacional del Centro de la Provincia de Buenos Aires (UNCPBA), GIB/CISAPA, Tandil 7000, Buenos Aires, Argentina; 5 School of Life Sciences, Shandong University, Qingdao 266237, China

**Keywords:** cutaneous vasoconstriction, infrared thermography, brown adipose tissue thermogenesis, cold-defensive behaviors

## Abstract

**Simple Summary:**

The present review aims to analyze the scientific evidence of the hypothalamic control of hypothermia and the central and peripheral changes that are triggered in domestic animals. After the perception of a core temperature decreases, several mechanisms aiming to prevent heat loss or produce heat are initiated. However, the type of mechanism and degree of activation depend on the species. Therefore, understanding the hypothalamic control of hypothermia could help us to understand the implications of the compensatory mechanisms in domestic animals.

**Abstract:**

When an organism detects decreases in their core body temperature, the hypothalamus, the main thermoregulatory center, triggers compensatory responses. These responses include vasomotor changes to prevent heat loss and physiological mechanisms (e.g., shivering and non-shivering thermogenesis) for heat production. Both types of changes require the participation of peripheral thermoreceptors, afferent signaling to the spinal cord and hypothalamus, and efferent pathways to motor and/or sympathetic neurons. The present review aims to analyze the scientific evidence of the hypothalamic control of hypothermia and the central and peripheral changes that are triggered in domestic animals.

## 1. Introduction

Thermoregulation is known as the maintenance of core body temperature in balancing heat loss and heat generation [1,2,3]. In mammals, an average of 37 ± 0.5 °C is considered a normal core temperature, and hypothermia is considered when the core temperature decreases to 35 °C [4]. Mild hypothermia is when the core temperature is between 32 and 35 °C, moderate from 28 to 32 °C, and severe from 24 to 28 °C [5]. The body temperatures of homeothermic animals are regulated by systems that utilize multiple behavioral and autonomic effector responses [6].

This response can differ according to the species. For example, between poikilothermic and homeothermic animals, significant thermoregulatory differences have been reported. It is known that poikilothermic animals cannot start the physiological mechanisms that homeothermic animals use to produce heat due to the lack of neural connections between the hypothalamus [7]. In studies where a cat’s brainstem was cut rostral to the mesencephalon, the animals had impaired thermoregulatory mechanisms, simulating poikilothermic activity [8]. Likewise, lesions in the posterior hypothalamus trigger failures in heat production/dissipation mechanisms [9], suggesting that, in poikilotherms, these regions do not have functional efferent pathways to the hypothalamus [10]. Ibraimov [11] mentioned chromosomal differences that make homeothermic animals more efficient at detecting subtle temperature changes. However, although it is mentioned that the central and peripheral receptors of poikilotherms are relatively insensitive to temperature changes, studies performed on lizards have shown that while warm-sensitive neurons in the preoptic area of the hypothalamus are less sensitive than those in mammals, cold-sensitive neurons are equivalent to homeotherms [12]. Moreover, hypothalamic oxytocin receptors have also been found in poikilothermic animals (tilapia) when exposed to cold stress, a similar response found in homeotherms, as well as increases in cortisol, a response associated with the metabolic changes that these species need to thermoregulate [13]. Poikilotherms resort to heat-seeking behavioral changes since they cannot initiate physiological and central mechanisms to produce heat [7]. Some authors refer that poikilotherms exhibit bradymetabolism rather than a deficient thermoregulation [14]; thus, they resort to other changes, such as glucose and lipid metabolism, to elicit secondary responses.

Nonetheless, several structures and events overlap with homeotherms, such as the importance of thermoreceptors to detect changes in external or internal temperature, as well as the role of the hypothalamus and the preoptic area in integrating these thermoregulatory mechanisms [15,16]. Although it is understood that physiological changes such as vasoconstriction occur in cutaneous blood vessels to reduce heat loss, other thermogenic events, such as shivering and non-shivering thermogenesis, as well as thermoregulatory behaviors are necessary for domestic animals to face hypothermia. The present review aims to analyze the scientific evidence of the hypothalamic control of hypothermia and the central and peripheral changes of thermoregulation in homeothermic domestic animals.

## 2. Search Methodology

A literature search was performed using databases such as the Web of Science, Scopus, Elsevier Science direct, and PubMed. The following keywords were used to select articles related to domestic animals and their responses against hypothermia, such as “livestock cold stress response”, “cold stress physiological changes”, and “behavioral changes and hypothermia”. Papers discussing the application of infrared thermography to detect changes in the surface temperature of domestic animals were also selected. As the inclusion criteria, the selected articles included relevant information about the thermoregulatory responses in domestic species, including cattle, water buffalo, pig, sheep, and dog studies. Some research including murine animal models was also included. Papers that did not assess the response of animals to cold stress or those that involved other species were not considered for the present review.

## 3. Hypothalamic Control of Hypothermia: From the Periphery to the Supraspinal Centers

### 3.1. Peripheral Thermoreceptor Activation

One of the main events that triggers the thermoregulation response is the activation of thermoreceptors [17,18]. These afferent neurons have ongoing activity at skin temperatures of about 10–42 °C [19,20]. The receptive terminals are located at the dermal–epidermal border or even in the epidermis [21]. In this regard, the most important thermoreceptor is the Transient Receptor Potential Menthol 8 (TRPM8). This receptor modifies the flow of ions associated with cold stimuli [22]. This response then allows the thermal stimuli associated with the cold to subsequently be transmitted to the central nervous system (CNS).

The response of TRPM8 can be induced by a wide range of cold temperatures. According to Zhang [22], this range includes temperatures below 26 °C—non-noxious cold—and thermal stimuli lower than 16 °C—noxious cold. Perhaps the importance of these receptors is represented by what was reported by Colburn et al. [23], who found that in TRPM8-knockout mice, in addition to lacking detectable levels of TRPM8 mRNA and protein, they also did not present behaviors such as icilin-induced jumping and cold sensations, as well as a significant reduction in injury-induced responsiveness to acetone cooling. Similar studies have shown that the null presence of these molecules limits the possibility of animals responding to thermal stress events associated with the cold [24,25,26]. Therefore, thermoreceptors sensitive to cold stimuli are needed to induce a physiological or behavioral response to cope with cold stress.

Kozyreva [27] explains that, in mammals, during a period of habituation to the cold, the proportion of low-frequency thermoreceptors associated with the cold (TRPM8), receptors that respond to temperatures between 24 and 25 °C, may decrease by 27%. However, when exposed to ambient temperatures between 28 and 30 °C, the expression of TRPM8 can increase up to 70% when compared to high-frequency receptors [28,29]. According to this change in the proportion of receptors, it is shown that TRPM8 detects the thermal stimuli associated with the cold, although it has been identified that TRPA1 can also contribute to cold signaling [30,31]. 

However, it is necessary to discuss that TRPM8 also contributes to the thermogenesis elicited by the brown adipose tissue (BAT). According to a review by Uchida et al. [32], TRPM8 receptors are also expressed in brown adipocytes, where it has been found that the activation of these by menthol up-regulates BAT TRPM8 receptors [33]. This shows that the activation of these receptors can increase the level of metabolic activity of this tissue to restore its thermoneutrality. In this sense, McKie et al. [34] evaluated the topical use of a pharmacological agonist mimetic of cold and TRPM8 receptors to stimulate BAT thermogenesis in three different strains of mice. They observed that infrared thermography was able to evaluate the administration of the agonist, recording a surface temperature increase of 1 °C in the interscapular region. Likewise, they found that there was an increase of 10% in oxygen consumption in adipose tissue.

These studies suggest the relationship between the thermogenesis response and the expression of thermoreceptors. Consequently, the scientific evidence shows the importance of thermoreceptors in the response to thermal cold stress since their activation has been shown to increase heat production via the BAT to restore euthermic body temperature.

### 3.2. Hypothalamus and Central Thermal Modulation

Once the thermal input is recognized by peripheral thermoreceptors, the information is transmitted from sensory afferents that innervate the skin and viscera to the spinal cord [19,35,36]. Ascending cold signals reach the dorsal horn of the spinal cord, where cold-sensitive neurons (CSNs) innervate the laminae (chief sensory nucleus of laminae V) [35,37]. Electrophysiological studies have shown that spinal neurons comprise warm-sensitive neurons (WSNs) and CSNs, as well as polymodal cells that can respond to noxious temperatures [33]. 

It has been described that most of the domestic mammals share a similar neuronal pathway to modulate their temperature. For example, pigs have myelinic fibers present in the BAT to transmit thermal information to hypothalamic centers [38,39]. As Babes et al. [40] mentioned, CSNs are those that respond to temperatures of 36 °C and 12 °C and are small-diameter myelinated Aδ-fibers in primates (with an average conduction of <30 m/s) and unmyelinated (C-) fibers in non-primate animals conducting at 0.5–2 m/s [21]. In rats, it has been reported that >70% of neurons in the dorsal root ganglion (DRG) are menthol-sensitive neurons [40], while in mice, 19% of neurons in the DRG and 45% of the superior cervical ganglia respond to cold stimuli [41]. Nonetheless, WSNs also participate in thermogenic responses when their activity is inhibited during hypothermia, and Nagashima et al. [6] mentioned that this mechanism might be the main hypothalamic control mechanism for heat production. 

Thermosensitive neurons send glutamatergic projections to the lateral parabrachial nucleus (LPB), specifically to the external lateral part of the LPB (LPBel) [37]. These inputs terminate in the midline of the preoptic area (POA) of the hypothalamus, known as the main thermoregulatory center of mammals [35,37]. The POA is located between the anterior commissure and the optic chiasm [42]. Neurons located in the medial preoptic area (MPOA) and medial preoptic nucleus (MnPO) respond to hypothalamic temperature as well as to thermoreceptive afferent signals from the spinal cord, blood, and viscera [4,43]. Studies have shown that cooling the POA elicits heat-promoting responses, such as vasoconstriction, brown adipose tissue (BAT) thermogenesis, and shivering [44]. 

Cold-defensive responses are triggered after the thermal input is processed in the MnPO. Consequently, a thermogenic afferent pathway involving the dorsomedial hypothalamus (DMH) and premotor neurons in the raphe pallidus (RPa) stimulates the autonomic, somatic, motor, endocrine, and behavioral changes [43,45]. These include cutaneous vasoconstriction to diminish heat losses and thermogenic mechanisms such as the use of brown adipose tissue [46,47]. On the one hand, sympathetic preganglionic neurons modulate BAT thermogenesis, while alpha and gamma motor neurons modulate shivering, and sympathetic premotor cutaneous vasoconstrictor neurons in the raphe cause vasoconstriction [48,49,50].

Thermal stimuli are also relayed from the thalamus to the somatosensorial cortex, where temperature perception and discrimination are integrated, motivating heat-preserving or heat-producing behaviors (discussed below) [35,43]. Neuroendocrine control is also involved in hypothermia-related defensive responses, where the main released hormones are adrenocorticotropic hormone, thyrotrophic hormone, growth hormone, follicle-stimulating luteinizing hormone, and prolactin [51]. Neurons in the medial ventral and lateral ventral POA are known to express the brain-derived neurotrophic factor (BDNF) and GABAergic properties, respectively [52]. Releasing thyroid hormones from the hypothalamus promotes heat production by increasing the metabolic rate [1]. In cattle, environments with a −15 °C temperature increase metabolic activity by 35% due to their increasing thyroxine levels [53]. Moreover, mild hypothermia (35 °C) in rats increases their plasma levels of corticosterone and thyrotropin-releasing hormone while their prolactin levels decrease [54]. Likewise, in cattle, exposure to mild/moderate (−1.05 °C) and extreme cold (−4.33 °C) resulted in increases in cortisol (7.00 ng/mL and 11.38 ng/mL, respectively) [55]. The hormonal response observed in hypothermic animals is greatly influenced by the hypothalamic–pituitary–adrenal (HPA) axis, which is triggered when the organism perceives alterations in homeostasis, including a decrease in their core temperature. For example, dairy cows exposed to a mean temperature of 3.4 °C and wind chills of −0.3 °C had higher plasma and fecal cortisol concentrations than control animals [56]. Moreover, the same animals showed behavioral alterations during cold periods, such as spending more time standing than lying down or eating. Similarly, in calves during the growing stage, Kim et al. [55] found that, when compared to threshold values, calves exposed to extreme cold stress (−4.33 °C) significantly increased their cortisol concentrations (8.97 vs. 12.60 ng/mL, respectively), while a decrease in blood glucose was registered (71.00 vs. 61.75 mg/dL, respectively). This was also observed by Uetake et al. [57], who determined that lactating dairy cows in cold-temperate climates had higher hair cortisol values (between 15 and 16 pg/mg of hair) than those located in warm-temperature regions (between 10 and 11 pg/mg). On the one hand, cortisol, adrenaline, and noradrenaline are known as heat-production hormones because they increase the level of lipid metabolism [58]. Cortisol release stimulates the immune system to restore homeostasis, increasing its concentrations under acute stress [59]. Moreover, adrenal hormones such as cortisol mobilize glucose, amino acids, and fatty acids because the body uses energy resources to maintain or initiate thermogenic mechanisms such as skeletal muscle growth to promote shivering thermogenesis [60], which is related to the decreased glucose concentrations [55].

**Figure 1:**
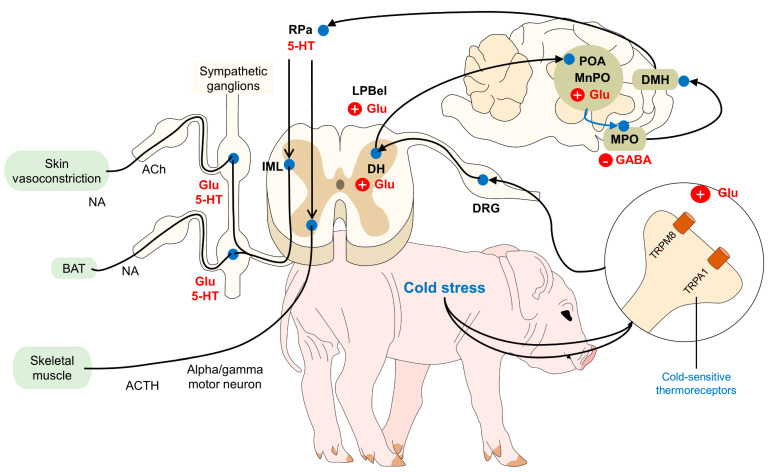
Hypothalamic control of hypothermia in domestic animals. The perception of low core body temperatures is due to the presence of thermoreceptors (TRPM8 and TRPA1). These receptors project to the DRG and DH in the spinal cord, where glutamatergic neurons transmit the thermal information to the POA. To generate thermogenic responses, connections from the POA to the PO, DMH, and rMR activate sympathetic or motor neurons that, in consequence, cause cutaneous vasoconstriction, BAT thermogenesis, or skeletal muscle shivering. Ach: acetylcholine; BAT: brown adipose tissue; Glu: glutamate; DH: dorsal horn; DMH: dorsomedial hypothalamus; DRG: dorsal root ganglion; IML: intermediolateral laminae; LPBel: lateral parabrachial nucleus lateral part; MnPO: median preoptic area; MPO: medial preoptic area; NA: noradrenaline; POA: preoptic area; RPa: rostral raphe pallidus; and 5-HT: serotonin.

Therefore, core body temperature is regulated by a negative feedback circuit, where the peripheral and hypothalamic pathways promote heat-conserving responses (Figure 1) [19]. Before discussing each of the thermogenic responses in mammals, it is important to mention that the activation of said mechanisms might differ according to the species. For example, while rodents can tolerate low body temperatures (18 °C), other mammals have a weak tolerance to body temperatures below 35 °C [61]. Neonatal piglets reach their lowest crucial temperature in environments at 34.6 °C; in contrast, for calves and lambs, this critical environmental temperature is set at 13 °C and 29 °C, respectively [62]. Therefore, the degree of the response might be influenced by the species.

## 4. Physiological Changes in Response to Hypothermia 

### 4.1. Vasomotor Response

**Figure 2:**
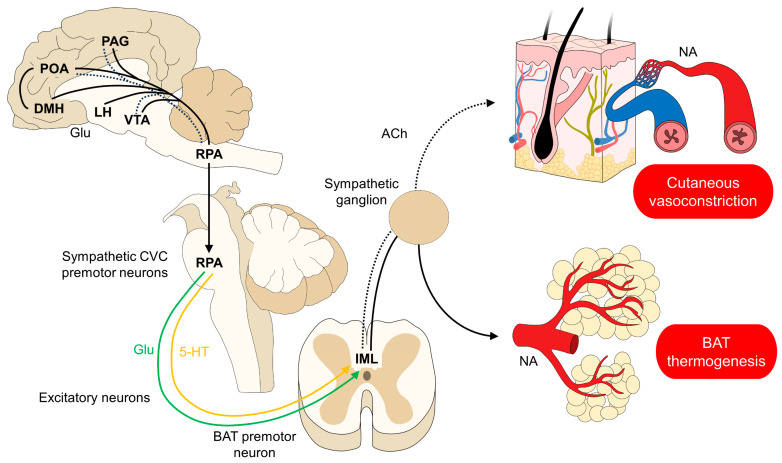
Modulation of cutaneous vasoconstriction and BAT thermogenesis. When the POA detects a decrease in core body temperature, two main responses are triggered. On the one hand, the neural pathway (marked with a non-continuous line) activates sympathetic CVC premotor neurons in the RPA to send glutamatergic and serotoninergic inputs to the IML. From these neurons, sympathetic axons innervating the endothelium of dermal blood vessels respond to NA release, eliciting vasoconstriction. On the other hand, the activation of premotor neurons innervating the BAT cause thermogenesis by the lipolysis of the BAT. ACh: acetylcholine; BAT: brown adipose tissue; DMH: dorsomedial hypothalamus; Glu: glutamate; IML: intermediolateral laminae; LH: lateral hypothalamus; NA: noradrenaline; PAG: periaqueductal gray; POA: preoptic area; RPA: rostral raphe pallidus; VTA: ventral tegmental area; and 5-HT: serotonin.

The vasomotor response refers to the modification in the diameters of cutaneous blood vessels to promote heat retention or loss (Figure 2). Verduzco-Mendoza et al. [47] explained that the two fundamental vasomotor responses in thermoregulation are vasodilation and vasoconstriction, which are mediated by cholinergic and noradrenergic innervations, respectively [63]. An example of this response is in anesthetized patients, as mentioned by Magnin et al. [64], who evaluated variations in the surface thermal response in eight healthy piglets undergoing anesthesia. They observed that as the anesthesia time progressed, a significant decrease in blood pressure of 11 mmHg coincided with a significant decrease of 660 cm^5^-m^2^ of systemic vascular resistance, as well as an increase of 41 mL-m^2^ of the global end-diastolic blood volume and a decrease of 1 °C in the thermal exchange gradient. The authors also found a negative correlation between blood pressure and the thermal exchange gradient (r = −0.40). These results suggest that the surface vasomotor responses affect the amount of heat dissipation through vasoconstriction and vasodilation. 

The changes in peripheral blood flow are an event that can be recognized with infrared thermography (IRT) since it is a method that detects small changes in blood circulation [65]. An example of this was presented by Jaén-Tellez et al. [66], who evaluated the correlation between the surface temperature assessed with IRT and the rectal temperature (RT) of weaned rabbits of Spanish common. A positive and moderate correlation was found between the ocular, ear, and nose surface temperature with RT (r = 0.39–0.49, *p* < 0.0001). However, the highest correlation values were found for the ear surface temperature. This is similar to what Schmitt and O’Driscoll [67] reported in piglets. By assessing the temperatures of the ear base, tip, and back, a positive and strong correlation was found between the ear base and RT (r = 0.86), while a moderate correlation was registered between the tip of the ear and RT (r = 0.53–0.59). Therefore, the ability of IRT to detect changes in the peripheral vasomotor mechanism suggests that IRT could be used to study hypothermia in domestic mammals. 

**Figure 3:**
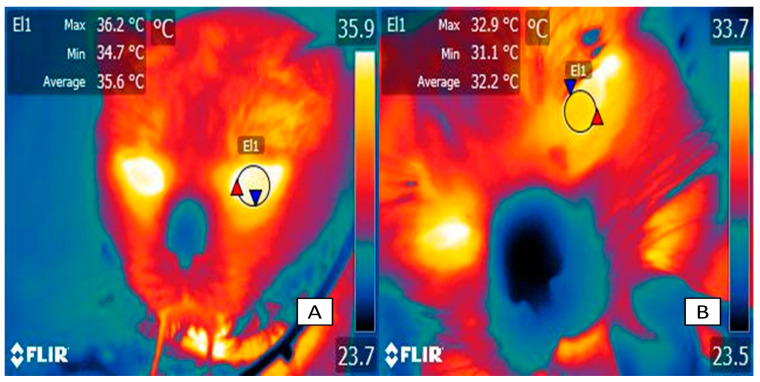
Thermal responses associated with the administration of general anesthetics. (**A**). The surface temperature of a male European domestic cat before anesthetic management for an advanced imaging diagnostic procedure is shown. The surface temperature at the periocular level (El1) recorded a maximum temperature of 36.2 °C, an average of 35.6 °C, and a minimum of 34.7 °C. (**B**). After the induction of anesthesia with propofol and maintenance with isoflurane, the temperature at the average periocular temperature (El1) decreased up to 3.6 °C. The decrease in surface temperature is due to the vasodilation that the anesthetic causes, allowing the redistribution of temperature. The maximum temperature is indicated with a red triangle, and the minimum temperature is indicated with a blue triangle.

Figure 3 shows the effect of inhalation anesthetics on surface temperatures in cats. In this sense, as mentioned by Picker et al. [68], inhalational anesthetic agents activate cardio-inhibitory neurons in the brainstem, increasing the level of vagal activity and triggering cardio-depressor and vasomotor activity. Anesthetics cause the vasodilation of cutaneous blood vessels, which increases the level heat loss, an effect observed in Figure 3.

**Figure 4:**
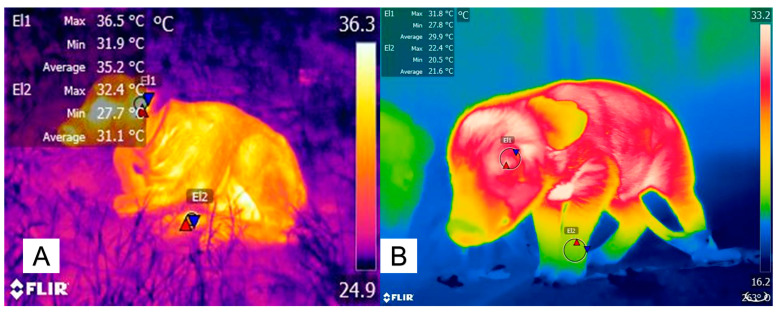
Differences between the surface temperatures of the peripheral and central regions in newborn animals. The differences in the surface temperature found between the central region (periocular, El1) and the peripheral region (thoracic or pelvic limb, El2) in both water buffaloes (**A**) and newborn piglets (**B**) are shown. In the case of the newborn water buffalo, an average difference in the temperature between both regions was set at 4.1 °C. In the piglet, an average difference in the maximum temperature of both regions was set at 9.4 °C. The possible explanation for this fact is that, during the perception of cold, the sympathetic postganglionic fibers release adrenaline and norepinephrine, which act on the α-2 adrenergic receptors present in the endothelium. This causes the vasoconstriction of the peripheral blood vessels that reduce the level of heat loss, preserving the temperature in central areas such as the head and thorax. The maximum temperature is indicated with a red triangle, and the minimum temperature is indicated with a blue triangle. Radiometric images were obtained using a T1020 FLIR thermal camera. Image resolution: 1024 × 768; up to 3.1 MP with UltraMax. FLIR Systems, Inc. Wilsonville, OR, USA.

**Figure 5:**
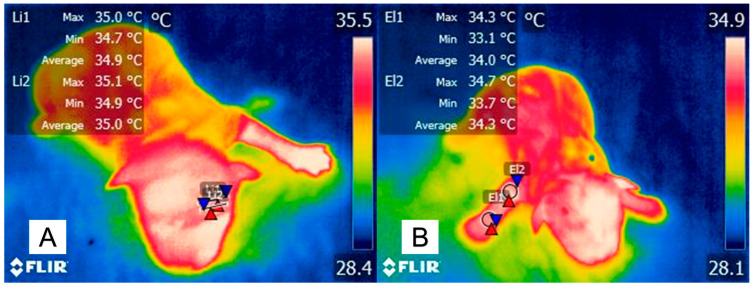
Comparison of surface temperatures in a newborn dog. (**A**) Central thermal windows. The upper (Li1) and lower (Li2) eyelids marked by a line are the central windows that are used to evaluate the thermal states of newborn puppies. The maximum temperature of these regions is 35.1 °C. In contrast, the (**B**) thoracic limb metacarpal (El1) and elbow (El2) thermal windows show lower temperatures by up to 0.8 °C. The differences in temperatures can be attributed to the immature thermoregulatory mechanisms in newborn dogs, which forces the organism to elicit changes to prevent heat loss (peripheral vasoconstriction) and produce heat (motivation to consume colostrum). The maximum temperature is indicated with a red triangle, and the minimum temperature is indicated with a blue triangle.

Although the previous examples used anesthetics to understand the vasomotor response, this event could help us to comprehend how the change in diameter of the capillary influences the temperature of peripheral regions. This effect could be explained by thermal inertia, meaning the variability in the capacity to reach a balance in core temperature when they are exposed to environmental conditions [69]. Although the meaning of thermal inertia is often applied to a physical object [70], the capacity to reach said balance might be affected by the disposition of blood capillaries in the different anatomical regions that can be considered thermal windows, as shown in Figure 4 and Figure 5.

These results show that between the peripheral regions and the central regions, there is a different thermal response mediated by the thermoregulatory hypothalamic pathway [71,72]. The release of catecholamines causes the vasoconstriction of blood vessels [73]. This event reduces heat exchange with the medium as a result of heat radiation. The general anesthesia model could help complement this explanation because this concept maintains that the decrease in temperature occurs in three phases. In the first phase, there is a decrease in core temperature, which is distributed in colder areas of the body, such as the skin, due to peripheral vasodilation. Subsequently, the central body temperature decreases linearly, and then this decrease in temperature progresses [74]. 

On the other hand, the effect that body weight has on the thermoregulatory ability of animals has also been discussed by some authors. In Napolitano et al.’s [75] study, where they monitored the surface thermal responses of 109 buffalo calves for six days after calving, they found that the temperature of the periocular regions was 1.3 °C higher in animals with a higher birth weight compared to animals with low weights at birth. However, they also observed a difference of up to 3 °C between the surface temperature of the eye and the one registered in the limbs. This is similar to what was reported in piglets, in whom low-birth weight animals had lower temperatures at the base of the ears (−1.3 °C) than normal birth weight piglets [67]. Moreover, the RT of low-birth weight piglets was also 1 °C below than high-birth weight animals. According to these results, it could be seen that birth weight influences both the core and surface temperature of animals, as well as the thermoregulatory mechanism that mammals will induce when facing cold stress. 

In the case of cold stress, vasoconstriction arises to reduce heat loss [76]. Thus, vasoconstriction has a key role in redistributing the temperature throughout the body. The above explanation coincides with what was observed by Sharma et al. [77], who evaluated the surface temperature of different regions, such as the back, rump, head, ear, forelegs, and hind legs, during the day in twelve Sahiwal heifers. They found that the surface temperature of the limbs was 5 °C lower compared to the rest of the regions. They also observed that the ambient temperature had a strong positive correlation with the surface temperature of all regions (r = 0.8). This was similar to what was reported by Vicente-Perez et al. [78], where they evaluated the relationship between the surface temperature of different regions with the rectal temperature, birth weight, and climatic variables in hair-breeding newborn lambs during the early spring season. They found a positive correlation between the environmental temperature and temperature humidity index with the surface temperature (0.69 ≤ r ≤ 0.82). Interestingly, a principal component analysis showed that between 50 and 60% of the variation of all body surface temperatures was explained by the temperature humidity index, but only 33% of the eye temperature. Although both studies suggest the close relationship between surface temperature variations and climatic variables in neonatal lambs, the fact that eye temperature has little explanation for climatic variables may reaffirm the idea that vasomotor changes help in redistributing the temperature to conserve heat in body regions with high metabolic compromise, as has been observed in species such as pigs and water buffaloes [75,79,80].

These studies help us to understand that vasomotor mechanisms play an important role in achieving thermal neutrality and that IRT could be implemented as an additional tool to recognize hypothermia in domestic animals. In this sense, Villanueva-García et al. [43] mentioned that assessing the animal’s surface temperature is a valuable tool that could help in identifying when the core temperature decreases, particularly in newborns. Moreover, surface temperatures could also estimate the RT in a non-invasive way, as reported by several authors who have found positive correlations between both parameters [81,82,83]. Thus, IRT might help us to not only recognize hypothermia but also act as a less invasive method to estimate the RT.

The vasomotor response is a primary mechanism that intervenes in heat loss due to the reduction in flow in peripheral blood vessels through vasoconstriction. This mechanism possibly also allows the redistribution of heat in the organism, which allows its conservation in regions with high metabolic commitments, such as the head and thorax, which would explain the differences in the thermal response across different regions.

### 4.2. Shivering

**Figure 6:**
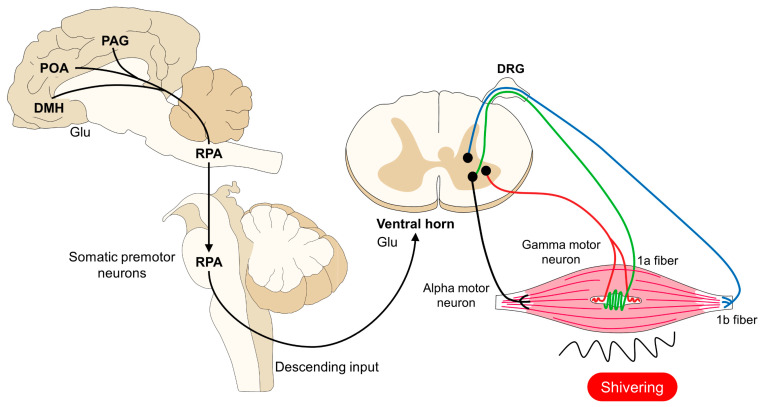
Central and peripheral control of shivering thermogenesis. Similar to the vasomotor and BAT thermogenesis, the POA, DMH, PAG, and RPA modulate muscular contractions. In the RPA, premotor neurons respond to hypothermia by activating alpha and gamma neurons that directly innervate muscle fibers. The sustained muscular contraction produces heat to promote an increase in core body temperature. ACh: acetylcholine; DMH: dorsomedial hypothalamus; DRG: dorsal root ganglion; Glu: glutamate; IML: intermediolateral laminae; PAG: periaqueductal gray; POA: preoptic area; and RPA: rostral raphe pallidus.

Shivering, like the vasomotor response, can be considered a local thermoregulatory mechanism that produces heat through the repeated contraction of muscles (Figure 6) [84,85]. Yang et al. [86] mentioned that the skeletal muscle plays a crucial role in the active mechanisms of thermoregulation in mammals, particularly in pigs. These authors compared the responses to cold exposure (4 °C) in cold-tolerant Tibetan pigs and cold-sensitive Bama pigs for 3 days. They found a significantly higher transcriptional response in the skeletal muscle of Tibetan pigs upon cold stimulation. In addition, in this breed, it was observed that the mitochondrial β-oxidation-related genes were positively regulated with the cold. Possibly, these results confirm that this tissue plays a relevant role when exposed to the cold by being able to help in generating heat using metabolically active substrates such as glucose or fatty acids. 

In horses, it was reported that involuntary muscle contractions with a frequency of 10 to 20 seconds do not fulfill any function other than the production of heat during hypothermia. Mejdell et al. [87] mentioned that shivering alone can increase, by up to 5-fold, heat production in a few seconds. In Icelandic horses, shivering was only observed when animals were exposed to 5 °C with heavy rain, in contrast to those animals maintained at −30 °C [88]. Similarly, Jørgensen et al. [89] found, in 22 mature horses, that shivering rarely occurred on winter days with temperatures above 0 °C. Despite this, although it is an efficient mechanism for heat production, it has a high energy cost [86]. This was studied by performing transcriptomic analyses of the skeletal muscle of Min pigs under chronically low temperatures. In this study, it was found that between 73 and 102 genes were positively regulated against oxidative stress, the nervous system, and lipid metabolism [90]. According to these authors, this suggests a genetic basis that helps us to understand the resilience of some species towards cold thermal stress.

The evidence shows that this genetic basis can establish a relationship between shivering and the increase in the consumption of energy resources, such as glucose and lipids, which facilitate the maintenance of muscle contractions. However, the metabolic cost of shivering is a challenge, mainly for newborn animals who have limited energy resources. Thus, some studies highlight the importance of colostrum or glucose supplementation to face hypothermia in newborns [79,91,92,93]. 

Another element that might be related to shivering is the characteristics of the fur. In this regard, Meisfjord Jørgensen et al. [94] evaluated the coat characteristics, body condition, and surface temperature in different body regions of 21 adult horses. They observed that the highest temperature was in the chest (22.5 ± 0.9 °C) and shoulders (20.4 ± 1.1 °C). They also observed that the animals with the greatest amount of fur had the lowest surface temperature. This suggests that the coat provides heat retention, and the presence of hair might be considered as an insulator that could prevent the use of thermogenesis mechanisms that require a significant metabolic cost [95]. Consequently, thermogenesis by shivering is an active mechanism for compensating hypothermia in domestic animals; however, because it consists of sustained contraction of muscle tissue, this mechanism largely depends on the availability of energy sources such as glucose to maintain it. 

### 4.3. BAT Non-Shivering Thermogenesis

Thermogenesis is known to be a process of heat production through the cellular dissipation of energy, which is mainly performed in the BAT (Figure 2) [96]. The BAT is a sympathetic, innervated thermogenic organ with a high number of mitochondria [97,98,99,100,101,102], with an average of 50–100 times more mitochondria than that of other types of adipose tissue [103]. Inside the mitochondria, the adipocytes have considerable amounts of uncoupling protein 1 (UCP1), which are receptors that create a proton leak in the mitochondrial membrane, diverting protons away from phosphorylation or oxidative phosphorylation processes—responsible for ATP synthesis—resulting in heat dissipation without energy storage [96,97,98,104,105,106]. 

The activation of the BAT’s thermogenic response is mediated by cold-related signaling perceived by the MnPO and inhibition of the MPA neurons [48]. Cooling the POA has been shown to increase the temperature of the BAT due to the inhibition of WSNs [6]. Glutamatergic projections activate sympathetic premotor neurons located in the raphe pallidus area (RPA) [96]. These neurons synapse in the intermediolateral cell column (IML) of the spinal cord, where nerve endings release norepinephrine (NE) [104,107,108]. The BAT contains β_3_ adrenergic receptors that are activated by NE and, consequently, activate UCP1 receptors to produce heat [48,96]. Concentrations of NE up to 10-fold have been found in the perirenal BAT of newborn lambs [109]. Moreover, heat production is also facilitated by increasing BAT vascularization, maintaining the metabolic supply of BAT to thermoregulate [110].

The amount of BAT (an average of 8–24 g in mammals), its triglyceride content (0.40–0.80 mg), and the efficacy of heat production depend on the species and even the breed since BAT development changes accordingly [98,111]. For example, mice and rats have large deposits of BAT in the interscapular region, while adult species such as sheep have brown adipocytes combined with white adipose tissue because skeletal tissue has a bigger role for thermoregulation [96]. Smith et al. [112] reported differences in the BAT between Angus and Brahman calves. While both breeds had perirenal adipocytes, Brahman BAT had two-to-three times more β_3_ adrenergic receptors than Angus calves. Moreover, only the BAT adipocytes in Brahman animals shrank after 48 h of exposure to 4 °C, which might suggest that this breed might exhaust its BAT reserves shortly after birth to adapt to cold stress. Similarly, perirenal BAT was reduced in newborn goats exposed to 6 °C, increasing UCP1 expression and triglycerides around 0.8-fold [113].

Graña-Baumgartner et al. [110] reported, in newborn Romney lambs, that exposure to an ambient temperature of 4 °C significantly increased the abundance of lipid classes such as glycerolipids. Likewise, changes in the morphology of BAT in newborn lambs were observed in animals raised at 3 °C, with smaller fat locules than animals raised at 26 °C. Furthermore, a rapid depletion of BAT was registered during the first two/three weeks of life [114]. In the case of cattle, for Hereford × Friesian calves, BAT corresponds to approximately 2% of the body weight of newborn calves, and BAT is mainly located in the intestinal, abdominal, pericardial, perirenal, prescapular, and orbital regions [115]. In contrast, pigs lack functional UCP1 proteins, making them a species susceptible to hypothermia [96]. Berg et al. [116] mentioned that pigs have mutations for the UCP1 coding sequence, inactivating the thermogenic capacity of the receptor and, therefore, impeding NST.

In this sense, IRT could help to recognize when animals are using their energy reserves to thermoregulate. For example, Labeur et al. [117] evaluated the effect of heat loss in lambs exposed to cold stress and the usage of subcutaneous brown fat depots. When comparing lambs born from shorn ewes and those in the control group (mock handling) exposed to a cold challenge, the authors found that lambs from the second group had higher core temperatures (+5 °C). In contrast, shorn lambs had higher surface temperatures, particularly at the hips, where the values were 1 °C above those recorded in control lambs. These results suggest that lambs born from cold-stressed ewes might have a higher thermoregulatory capacity due to the increased BAT deposition. This is similar to what was reported by other authors, who mention that BAT deposits are particularly located at the hips and in the interscapular space [36,118,119]. 

Therefore, IRT is known as a tool that recognizes changes in peripheral blood circulation. However, it is important to note that IRT can also help to identify thermogenesis when mammals are exposed to cold stress by assessing the surface temperature of regions where BAT deposits are located.

## 5. Behavioral and Postural Reactions of Animals to Face Hypothermia

The connection between the hypothalamus and the somatosensory cortex results in discriminatory responses that elicit behavioral and postural changes in animals to prevent heat loss or enhance heat production [19,21,36]. According to the species, several behavioral changes can be observed. For example, Kim et al. [55] evaluated the behavioral response of Korean native calves and steers under mild/moderate (−1.05 °C) and extremely cold (−4.33 °C) conditions. Both groups increased the standing time when exposed to extreme cold (316.25 and 261.88 min/day, respectively) compared to the threshold (251.88 and 232.50 min/day, respectively), while the lying time was significantly reduced (283.75 and 338.13 min/day, respectively). In the same species, long-term cold stress (at −14.02 °C) increased the lying time (26.18 ± 0.948%) and feeding time (13.68 ± 0.281%) and reduced the standing (20.56 ± 0.731%), walking (3.75 ± 0.202%), and water drinking (0.76 ± 0.070%) of Simmental crossbred bulls [120]. Similarly, feedlot steers housed at −40–19 °C increased their ruminating (32%) and lying down behaviors (24%) [121]. 

**Figure 7:**
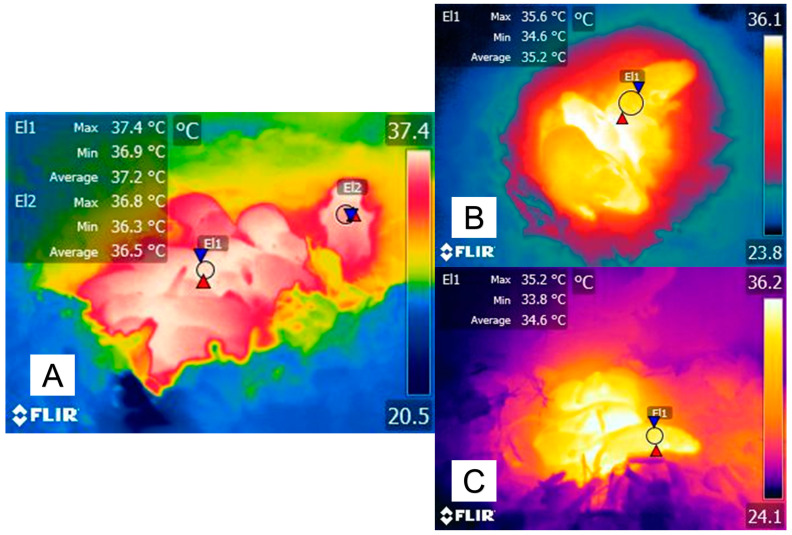
The importance of thermoregulatory behaviors and neonatal development in newborn rat pups. (**A**) The influence of huddling as a thermoregulatory behavior highly influences the thermal states of newborn rats during the first weeks of life. It can be observed that the maximum interscapular temperature of the pup in the center of the nest (El1) is 0.6 °C higher than the values recorded in a pup located in the nest periphery (El2). (**B**) shows the thermoregulatory advantage that hair growth has on the surface temperature of animals. In a 12-day-old rat pup with fur growth, the maximum temperature at the interscapular space (El1) is 35.6 °C. In contrast, in newborn pups (**C**), the maximum temperature of the same region is 0.4 °C lower, although both animals are inside the nest with their conspecifics. These thermal images help us to understand that behavioral adjustments play an essential role in mammal thermoregulation. The maximum temperature is indicated with a red triangle, and the minimum temperature is indicated with a blue triangle. Radiometric images were obtained using a T1020 FLIR thermal camera. Image resolution: 1024 × 768; up to 3.1 MP with UltraMax. FLIR Systems, Inc. Wilsonville, OR, USA.

The increased lying time can be regarded as a heat retention behavior similar to huddling in rabbits, as mentioned by Gilbert et al. [122]. In New Zealand × Californian rabbits, huddling delays the thermogenic response of BAT activation and prevents hypothermia during cold challenges at 18, 15, and 10 °C, particularly in non-insulated pups [122] (Figure 7). On the other hand, severe hypoxia in newborns is associated with a decrease in metabolic heat production, an effect that has been observed in other species such as piglets, lambs, calves, puppies, and kittens [123].

In sheep, increases in feeding have been reported as a way to meet the energy requirements to maintain an adequate core body temperature [124]. However, similar to what was observed in dairy goats, lying (49.8%) and feeding behavior (22.7%) were not affected by low temperatures (−10 °C), showing similar percentages to animals maintained at 10 °C [125]. Therefore, domestic animals resort to behaviors that maintain their core body temperature, which, together with their physiological responses, help them to thermoregulate during hypothermia. Moreover, Table 1 shows the physiological, endocrine, and behavioral changes that the main discussed domestic species have when exposed to cold stress.

## 6. Perspectives 

The evidence discussed so far shows that most domestic animals present peripheral mechanisms such as the vasomotor reaction; however, there are still areas of opportunity for study that would help to understand these mechanisms, for example, the relationship of the vasomotor response with birth weight because, as has been observed in both piglets and ruminants, those animals with low birth weight have greater difficulty compensating for temperature due to the availability of energy resources [75,139]. However, it is not clear whether this limited availability of energy substrate can also affect the vasomotor response, as has been observed in humans, where babies born with low birth weight present a poor vasoconstriction response. 

Understanding the hypothermia-related mechanism of domestic animals to produce heat or prevent heat loss can be (and should be) applied by veterinary practitioners even on real farms. For example, recording the behavior of livestock may help to recognize when an animal is standing/lying for prolonged periods to maintain body heat. This could suggest that the environmental conditions provided to the animals are not meeting their biological needs, including their thermal requirements. Although most of the research regarding stockpeople and livestock interaction is not focused on identifying cold stress, current research has shown that skilled personnel in identifying changes in animals’ body language can help to assess their health status or disease incidence [140,141]. 

Regarding the physiological parameters or vasomotor changes, these might be challenging to assess in field conditions. However, tools such as IRT could help to recognize hypothermia by assessing the surface temperature in different regions. Nonetheless, it is important to recognize that variations in peripheral blood flow and surface temperature—assessed through IRT—need complementary tools to precisely determine if the changes are due to thermal stress or to underlying causes such as inflammation or generalized sickness [142]. Currently, several studies have mentioned the use of IRT to identify inflammatory processes such as mastitis [143] or osteoarthritis [144], but also to febrile animals and those affected by infectious diseases [145,146]. The response and vasomotor change depend on the selected region where surface temperature is evaluated. Therefore, although IRT serves as a non-invasive method to detect blood flow alterations, other techniques and gold-standard methods are still required to evaluate both the thermal and health status of domestic animals.

On the other hand, regarding thermogenesis due to shivering, an important point to discuss is the relationship between the presence of hair and shivering, that is, as has been observed in horses, where the presence of hair limits the use of shivering because it prevents heat loss to the environment by conserving the hot air that comes into contact with the skin [87,94]. In this sense, another important aspect to address is whether the length of the hair can equally influence the thermal insulation formed by the hair coat as well as the type of coat, as has been seen when it influences the evaluation of infrared thermography [147,148]. 

Other aspects where IRT is being implemented but requires further analysis in several species are the color of the coat and the effect that this can have on an animal’s thermoregulation. For example, recently, Nejad and Lee [149] reported that multiparous dairy cows with black coats had lower hair cortisol and higher hair serotonin concentrations than cows with white coats, while serum cortisol and serotonin did not show significant differences. These results regarding blood parameters could be complemented with IRT to comprehensively understand the effect that external characteristics of domestic mammals have on their thermoregulatory capacity and how this could be implemented within management practices. 

## 7. Conclusions

In domestic animals, the main thermoregulatory responses to hypothermia are mechanisms to produce heat or prevent heat loss. Peripheral vasoconstriction is used to reduce heat loss, while shivering and non-shivering thermogenesis increase heat production. These mechanisms arise from the recognition of thermal input from thermoreceptors (e.g., TRPM8) and central processing in the hypothalamus. 

Together with these responses, behavioral changes aimed at maintaining heat (e.g., increasing the time spent lying down) or producing metabolic heat by increasing feed intake also contribute to maintaining animals core body temperature within normal ranges. Understanding the physiological consequences that cold-related pathways cause in the organism is an element of promoting thermal comfort in domestic animals and proposing alternatives to prevent hypothermia. 

## Figures and Tables

**Table 1:** Physiological, endocrine, behavioral, and surface temperature changes observed during cold stress in some of the species included in the present review.

Species	Physiological Changes	Endocrine Responses	Behavioral Changes	Surface Temperature Changes	References
Cows	↑ Heart rate ↑ Respiratory rate ↓ Blood pressure Shivering	↑ Plasma cortisol ↑ Fecal cortisol ↑ Hair cortisol ↑ T4 ↑ Non-esterified fatty acid ↓ Glucose	↑ Time standing ↑ Rumination ↑ Time lying down ↓ Grooming ↓ Dry matter intake	↓ Orbital ↓ Dorsal ↓ Head ↓ Shoulder ↓ Flank	[55,56,57,121,126]
Goats	↑ Heart rate ↑ Respiratory rate ↑ Pulse rate Shivering	↑ Plasma cortisol ↑ T4 followed by ↓ ↑ Glucose followed by ↓ AST ALT	↑ Time standing ↑ Walking ↑ Feed intake ↓ Water consumption ↓ Time lying down	↓ Interdigital space ↓ Coronary band ↓ Mid-head ↓ Nose ↓ Ear base	[125,127,128,129]
Sheep	↑ Heart rate ↑ Respiratory rate ↑ Pulse rate	↑ Plasma cortisol ↑ T3 ↑ T4 ↓ Glucose	↑ Time standing ↑ Rumination ↑ Feed intake	↓ Interdigital space ↓ Coronary band ↓ Mid-head ↓ Nose ↓ Ear-base	[130,131,132,133,134]
Pigs	↑ Heart rate ↑ Blood pressure ↑ Cardiac output	↑ ACTH ↑ Plasma cortisol ↑ T3 ↑ T4	↑ Search warm environment ↑ Huddling ↓ Locomotor activity ↑ Feed intake	↓ Full body ↓ Ear	[135,136,137,138]

↑: the parameter increases; ↓: the parameter decreases.

## Data Availability

Data sharing is not applicable.
